# Beyond the visible: preliminary evaluation of the first wearable augmented reality assistance system for pancreatic surgery

**DOI:** 10.1007/s11548-024-03131-0

**Published:** 2024-06-07

**Authors:** Hamraz Javaheri, Omid Ghamarnejad, Ragnar Bade, Paul Lukowicz, Jakob Karolus, Gregor Alexander Stavrou

**Affiliations:** 1 German Research Center for Artificial Intelligence (DFKI), Kaiserslautern, Germany; 2 Department of General, Visceral, and Oncological Surgery, Klinikum Saarbrücken, Winterberg 1, 66119 Saarbrücken, Germany; 3 MeVis Medical Solutions AG, Bremen, Germany; 4 University of Kaiserslautern-Landau, Kaiserslautern, Germany

**Keywords:** Augmented reality, Wearable devices, Surgical planning, Navigation system, Pancreatectomy

## Abstract

**Purpose:**

The retroperitoneal nature of the pancreas, marked by minimal intraoperative organ shifts and deformations, makes augmented reality (AR)-based systems highly promising for pancreatic surgery. This study presents preliminary data from a prospective study aiming to develop the first wearable AR assistance system, ARAS, for pancreatic surgery and evaluating its usability, accuracy, and effectiveness in enhancing the perioperative outcomes of patients.

**Methods:**

We developed ARAS as a two-phase system for a wearable AR device to aid surgeons in planning and operation. This system was used to visualize and register patient-specific 3D anatomical models during the surgery. The location and precision of the registered 3D anatomy were evaluated by assessing the arterial pulse and employing Doppler and duplex ultrasonography. The usability, accuracy, and effectiveness of ARAS were assessed using a five-point Likert scale questionnaire.

**Results:**

Perioperative outcomes of five patients underwent various pancreatic resections with ARAS are presented. Surgeons rated ARAS as excellent for preoperative planning. All structures were accurately identified without any noteworthy errors. Only tumor identification decreased after the preparation phase, especially in patients who underwent pancreaticoduodenectomy because of the extensive mobilization of peripancreatic structures. No perioperative complications related to ARAS were observed.

**Conclusions:**

ARAS shows promise in enhancing surgical precision during pancreatic procedures. Its efficacy in preoperative planning and intraoperative vascular identification positions it as a valuable tool for pancreatic surgery and a potential educational resource for future surgical residents.

## Introduction

Despite recent advancements in chemotherapy and surgical methods, the prognosis for pancreatic cancer remains poor, marked by a 5-year survival rate below 10% [1]. Surgical resection, specifically achieving complete eradication of the tumor (R0 resection), currently stands as the solitary curative strategy. Nevertheless, the inherent complexity of pancreatic surgery persists due to the retroperitoneal anatomy of the organ and its close proximity to the vital vascular systems. Notably, tumor arterial involvement has emerged as the principal impediment to achieving R0 resections. At the time of diagnosis, approximately 30% of patients present with locally advanced disease characterized by extensive vascular involvement [2]. Moreover, the variability in the origin of peripancreatic vessels poses an additional challenge, contributing to difficulties in anatomical identification and, consequently, increasing the risk of intraoperative complications. While the postoperative mortality rate in high-volume centers is currently below 5%, associated morbidities such as intraoperative blood loss and the requirement for blood transfusions remain notably high [3–5].

Hence, cautious preparation and dissection of the peripancreatic vessels during pancreatic surgery are imperative to reduce perioperative complications and enhance oncological outcomes. The incorporation of preoperative computed tomography (CT) images and augmentation of derived reconstructed 3D models of peripancreatic structures during surgical procedures may have significant potential in this particular domain. Augmented reality (AR)-based guidance systems have been utilized in recent decades, particularly in the fields of orthopedic, plastic, and neurosurgery [6–8]. Nevertheless, in contrast to these surgical fields, the evolution of AR-based guidance systems for abdominal surgery has been impeded by challenges such as organ shifting and deformities [9–11].

Given the retroperitoneal nature of the pancreas, which is characterized by minimal intraoperative organ shifts and deformations, pancreatic surgery is a promising candidate for AR-based systems. A limited number of studies have investigated the effect of AR-based systems during pancreatic surgery [12–16]. Of note, these investigations predominantly employed AR technology using 2D-display-based modalities, such as monitors, tablets, and smartphones, with mostly manual 3D model registration. The reliance on 2D-based methods without accurate automated registration introduces specific constraints that detrimentally affect spatial perception and necessitate manual manipulation by the operator. This circumstance has the potential to impede decision-making processes, thereby posing the risk of prolonged surgical duration and intraoperative complications.

We present the preliminary outcomes of our ongoing prospective study with the aim of investigating the usability, feasibility, and effectiveness of the designed and developed wearable AR assistance system, ARAS, during pancreatic surgery. To the best of our knowledge, this is the first prospective study to establish and enhance wearable AR-assisted surgery by implementing an accurate semi-automated intraoperative registration method specifically designed for pancreatic surgery.

## Methods

### Setting

The preliminary outcomes of the five patients included in the ARAS prospective study are presented in this manuscript. In this prospective study, 25 patients (> 18 years old) with underlying (borderline) resectable pancreatic tumors requiring any type of pancreatic resection will be included. The study protocol was approved by the Ethics Committee of the Medical Association of Saarland (registration number: 159/23). This prospective study was registered at ClinicalTrials.gov under the registration number NCT06208579. All the procedures were performed in accordance with the most recent revision of the Declaration of Helsinki.

### System design

#### Patient-specific 3D anatomical model reconstruction

The 3D reconstruction of the patient’s vascular anatomy and tumor was achieved through the utilization of the patient’s most recent (< two weeks) three-phase, contrast-enhanced, multidetector CT scan using the MeVis Liver Suite software (MeVis Medical Solutions AG, Bremen). An expert surgeon performed the reconstruction process. Accordingly, 3D models of the tumor, xiphoid process of the sternum, portal vein, splenic vein, superior and inferior mesenteric veins, inferior vena cava (with left renal vein entry), celiac axis (hepatic, gastric, and splenic arteries), gastroduodenal artery (GDA), and superior mesenteric artery (SMA) were extracted.

#### ARAS

ARAS was designed and developed for pancreatic surgery through an iterative process driven by the collection of qualitative data gathered through extensive interviews with expert surgeons. The system comprises two essential components: a software application tailored for wearable AR devices and a marker set. The software was designed and developed using the Unity 3D game engine 2020.X (Unity Software Inc., San Francisco, California, USA) for Microsoft HoloLens 2 (Microsoft Corporation, Redmond, Washington, USA).

The ARAS software is structured into two distinct phases: pre- and intraoperative. The preoperative session served the purpose of surgical planning by offering a manipulatable and scalable 3D model of patient-specific reconstructed peripancreatic structures for better preoperative observation. Moreover, the system provided the capability to perform resections on the 3D model, which was also used to demonstrate and discuss the surgical plan with other participating surgeons, especially for education of inexperienced surgical residents.

The intraoperative session was designed to register these 3D models during the surgical procedure, while preserving the actual size and topography of the structures. Additional features, such as CT images and patient history visualizers, were also implemented in both the pre- and intraoperative phases. The system was designed to be fully controllable through natural communication skills, leveraging large language models, generative AI, and voice inputs to ensure a minimal cognitive load on operators during surgical intervention.

A marker-based AR approach was employed to ensure accurate intraoperative registration of patient-specific 3D anatomical models. This approach involves the use of specially designed visual markers as triggers for AR-content overlays. The system recognizes and tracks these markers in real time using the device camera, enabling the accurate alignment of virtual objects with the physical world. Once a marker is detected, the system can superimpose digital content, such as 3D models or information on the marker’s location from the user’s viewpoint.

ARAS employs a dual-marker approach, along with two distinct tracking methodologies for progressive registration during the surgical procedure. Both markers were fabricated using 3D printing technology, which enables sterilization for use in the surgical environment.

**Fig. 1:**
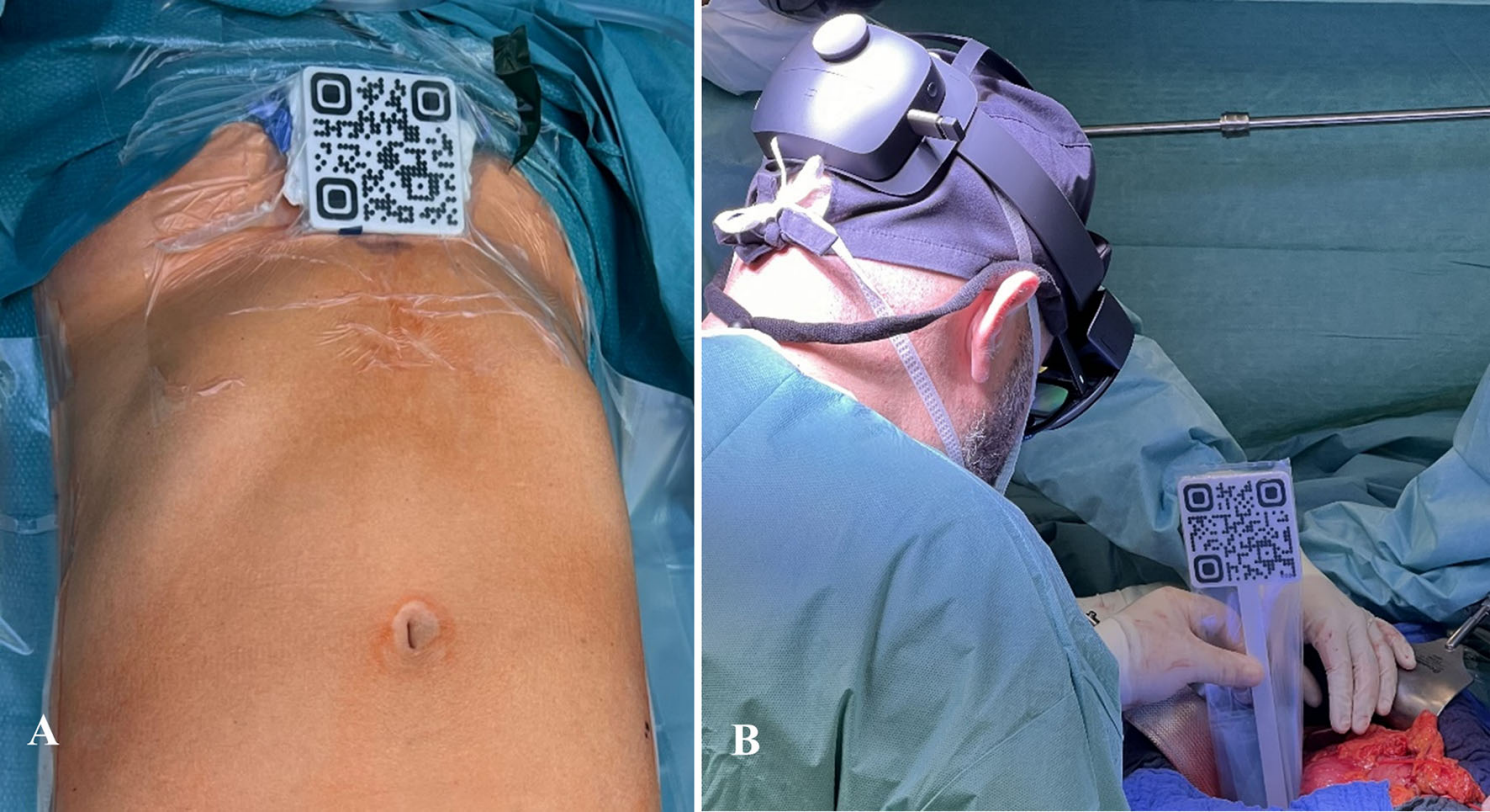
**a** Preparation of the surgical field includes the fixation of a sterilized marker onto the xiphoid process before the laparotomy and **b** marker-based tracking using the entry of left renal vein into the inferior vena cava as the registration point

1. Sternum marker and active tracking: The orientation and position of the marker on the skin surface relative to the xiphoid process were precisely measured from CT images. These measurements were then used to determine the positions of the 3D models relative to the marker. Initially, a surgeon identified the xiphoid process before laparotomy and affixed the predesigned and sterilized marker to it (Fig. 1a). The marker attached to the xiphoid process of the sternum was used to register patient-specific anatomical models. This marker was actively tracked during the initial preparation phase of the operation, and the 3D model adjusted its position in real-time according to the movement of the marker. This strategy was adopted to ensure precise registration due to minimal mobility during surgery, as the active tracking of the sternum marker provided accurate positional information and mitigated registration errors induced by the patient’s respiratory movements.
2. Vena cava marker and passive tracking: As the abdominal region was exposed, the transition to the entry of the left renal vein into the inferior vena cava as the second registration point was made. The selection of this registration point was motivated by its inherent stability in relation to other vascular structures, maintaining topographical consistency, and remaining unaffected by any movement. Owing to spatial constraints and potential interference with the surgical procedure, active tracking of the vena cava marker is considered impractical, leading to the adoption of a passive tracking method. The passive tracking method involves registering the 3D model using a predesigned marker, and then freezing the model position using additional features captured from the user’s field of view. The positioning of the vena cava marker was performed by the surgeon during surgery by identifying a predetermined registration point on the inferior vena cava. The vena cava marker was designed as a legged marker with a fixed leg length to facilitate its positioning above the abdominal area, without the necessity of directly attaching the marker to the vena cava (Fig. 1b). This design allowed precise registration of the inferior vena cava while maintaining an optimal spatial arrangement during the surgical procedure.

### Outcome measures

**Fig. 2:**
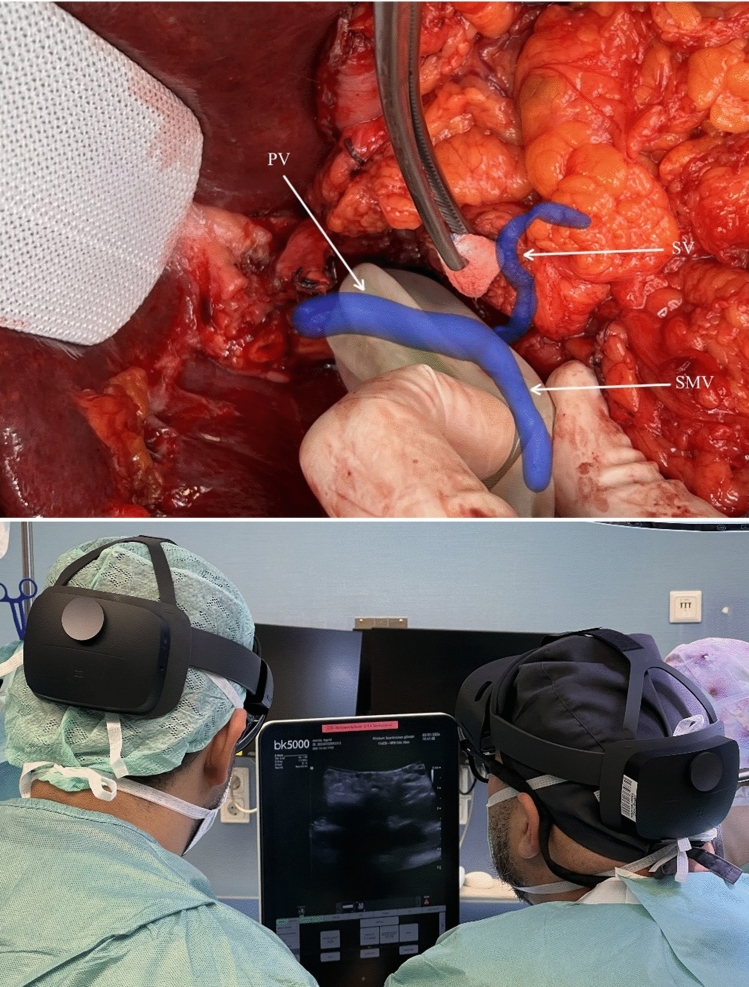
Intraoperative evaluation of the location and precision of the registered 3D anatomy using ultrasonography. Portal vein, PV; splenic vein, SV; superior mesenteric vein, SMV

The operative strategy was preoperatively discussed and planned using ARAS by four surgeons who intended to attend the surgery. ARAS was consistently utilized intraoperatively by two surgeons, while the other two surgeons employed ARAS as needed during the critical phases. During surgery, the location and precision of the registered 3D anatomy were evaluated by assessing the arterial pulse and using Doppler and duplex ultrasonography (Fig. 2).

An assessment of the usability, accuracy, and overall effectiveness of ARAS for both pre- and intraoperative sessions was also performed by four surgeons, who participated in the surgeries, using a five-point Likert scale questionnaire (Table 1). Ultimately, a total of 20 questionnaires (corresponding to five patients) were evaluated. Continuous variables were presented through means ± standard deviations, alongside median values and ranges, were visually represented through box plot illustrations.

**Table 1:**
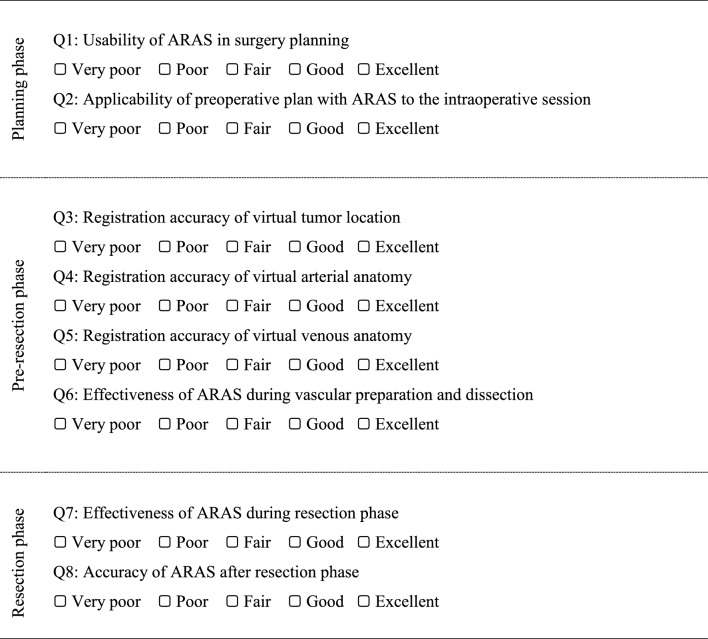
Assessment of ARAS during different phases of the operation

CT: computed tomography; ARAS: augmented reality-assistance systems

In addition, any changes or modifications to the planned strategy during the surgery were documented. Intraoperative blood loss, operative time, resection margin, perioperative complications, and duration of hospitalization were also documented.

## Results

Baseline demographic and perioperative clinical data of patients are presented in Table 2.

**Table 2:** Demographic and perioperative clinical data of patients

Patient no	Age (years), sex	Preoperative diagnosis	Operative procedure	Blood loss (ml)	Intraoperative complications	Operative time (min)	Perioperative ARAS-related complications	Hospitalization (days)
1	48, F	Borderline resectable pancreatic body cancer	EDP-CAR	550	None	320	None	9
2	82, F	Cystic neoplasm	Extended distal pancreatosplenectomy	50	None	125	None	7
3	73, F	Cystic neoplasm	Extended distal pancreatosplenectomy	50	None	140	None	7
4	59, M	Uncinate process cancer	PPPD	350	None	310	None	10
5	69, F	Uncinate process cancer	PPPD	400	None	280	None	12

ARAS: augmented reality-assistance systems; EDP-CAR: extended distal pancreatosplenectomy with en bloc celiac axis resection; PPPD: pylorus preserving pancreaticoduodenectomy; F: female; M: male

### Patient 1 (Appleby procedure)

**Fig. 3:**
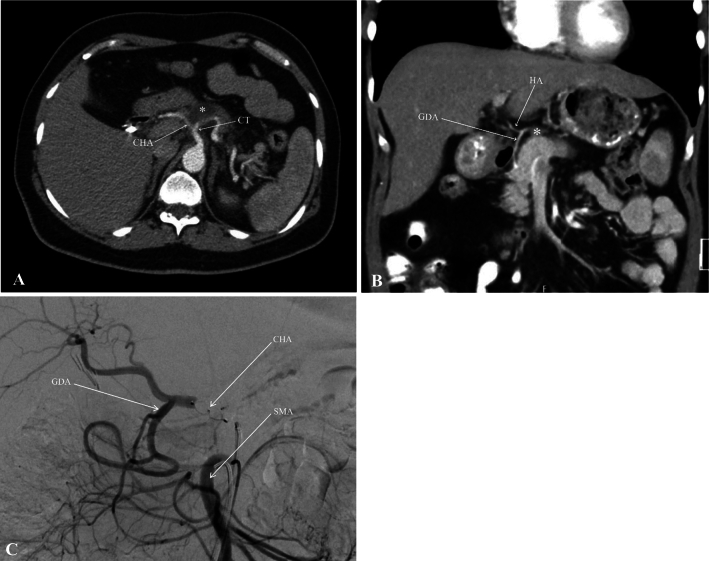
**a** Borderline resectable pancreatic body cancer (*), infiltrating the celiac trunk (CT) and common hepatic artery (CHA); **b** CHA tumor infiltration (*) and blood supply via gastroduodenal artery (GDA); **c** the figure illustrating angiographic closure of the CHA and reversal blood supply of hepatic artery (HA) via superior mesenteric artery (SMA) and GDA

**Fig. 4:**
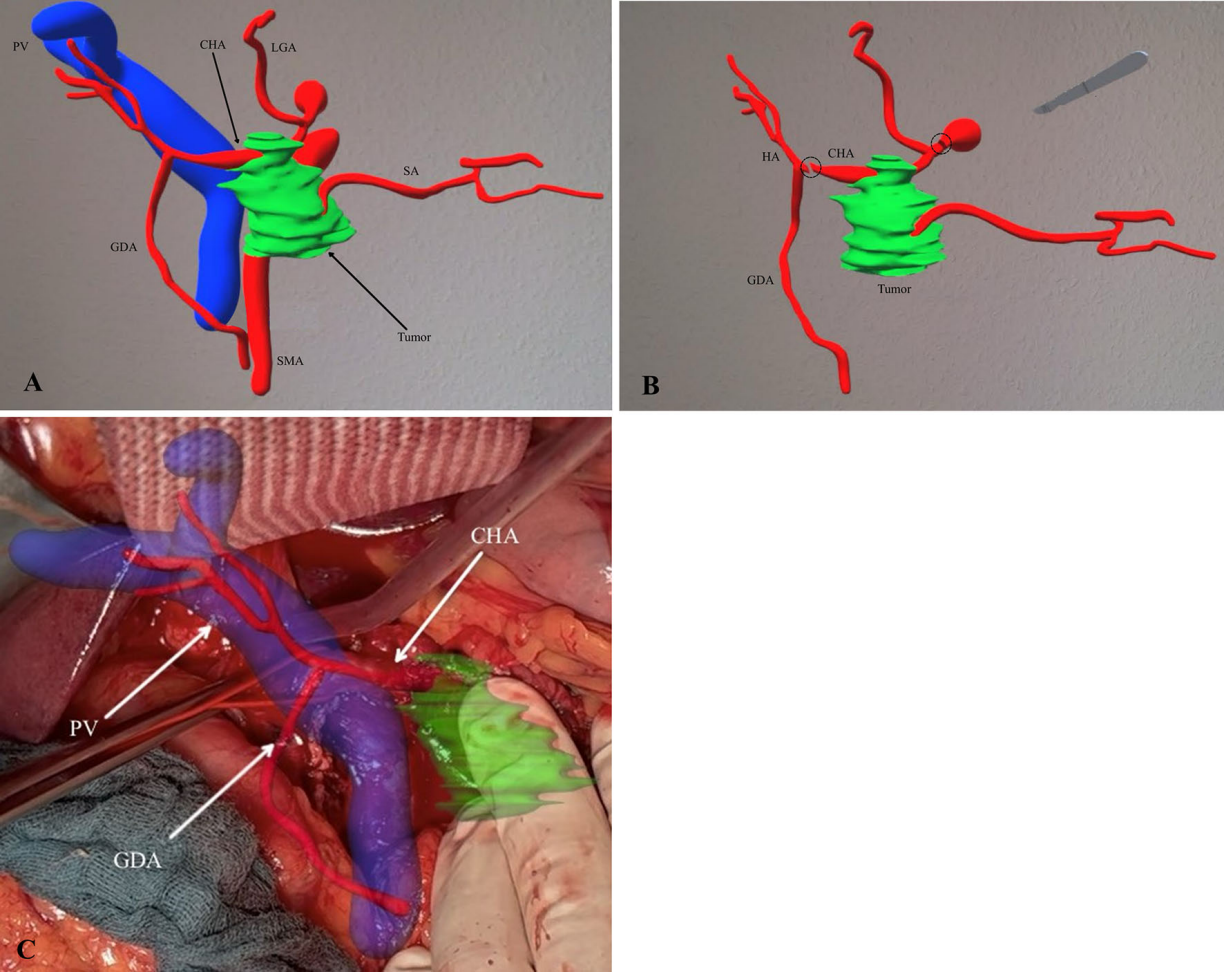
**a** Reconstructed 3D model of tumor with peripancreatic vascular system; **b** illustration of planned procedure as extended distal pancreatosplenectomy with an en-bloc resection of the common hepatic artery (CHA) and celiac axis; **c** intraoperative snapshot during vascular preparation before resection phase using ARAS. Exposition and marking the CHA (red vascular loop). Gastroduodenal artery, GDA, splenic artery, SA; hepatic artery, HA; portal vein, PV; superior mesenteric artery, SMA; left gastric artery, LGA

A 48-year-old female was diagnosed with borderline resectable pancreatic body cancer infiltrating the celiac axis and the common hepatic artery (Fig. 3a and b, Stage III: cT4 cN1 cM0). Because of the tumor’s complex location necessitating an extended distal pancreatosplenectomy with en bloc celiac axis resection (Appleby procedure), the patient underwent neoadjuvant chemotherapy comprising eight cycles of the FOLFIRINOX protocol. Subsequent to chemotherapy, a marked regression of the tumor, as evidenced by posttherapy CT examination and tumor marker (CA 19–9 = 48 to 5 U/mL), was observed, with no sign of metastasis. In preparation for the surgical procedure, interventional closure of the common hepatic artery was performed, facilitating blood flow reversal via the SMA into the GDA, and subsequently into the hepatic artery (Fig. 3c). As shown in Fig. 4, the surgical approach was planned using ARAS.

The intraoperative registration of the 3D model proceeded seamlessly. The initial identification of the tumor and vascular system using ARAS was evaluated by attending surgeons via intraoperative ultrasonography, which demonstrated close similarity to the actual anatomy. Utilizing ARAS during the operation provided precise identification of vital vessels, including the hepatic artery, GDA, and SMA. ARAS facilitated secure dissection of the common hepatic artery and celiac axis, while ensuring preservation of the GDA and SMA (Fig. 4c). As per the preoperative plan, extended distal pancreatosplenectomy was performed along with en bloc resection of the celiac axis and a 4/5 gastrectomy. Post-resection Doppler and duplex ultrasonography revealed a compromised intrahepatic flow. Further exploration demonstrated hepatic artery dissection, prompting a patch plasty procedure between the GDA and the left and right hepatic arteries, using a bovine patch. Subsequently, ultrasonography revealed normal arterial flow.

Intraoperative parameters included a blood loss of 550 ml and operation time of 320 min. Histopathological examination confirmed pancreatic adenocarcinoma with a tumor-free resection margin (R0). The patient was discharged nine days postoperatively and remained alive without any signs of tumor recurrence at the three-month follow-up.

### Patient 2 and 3 (extended distal pancreatectomy)

**Fig. 5:**
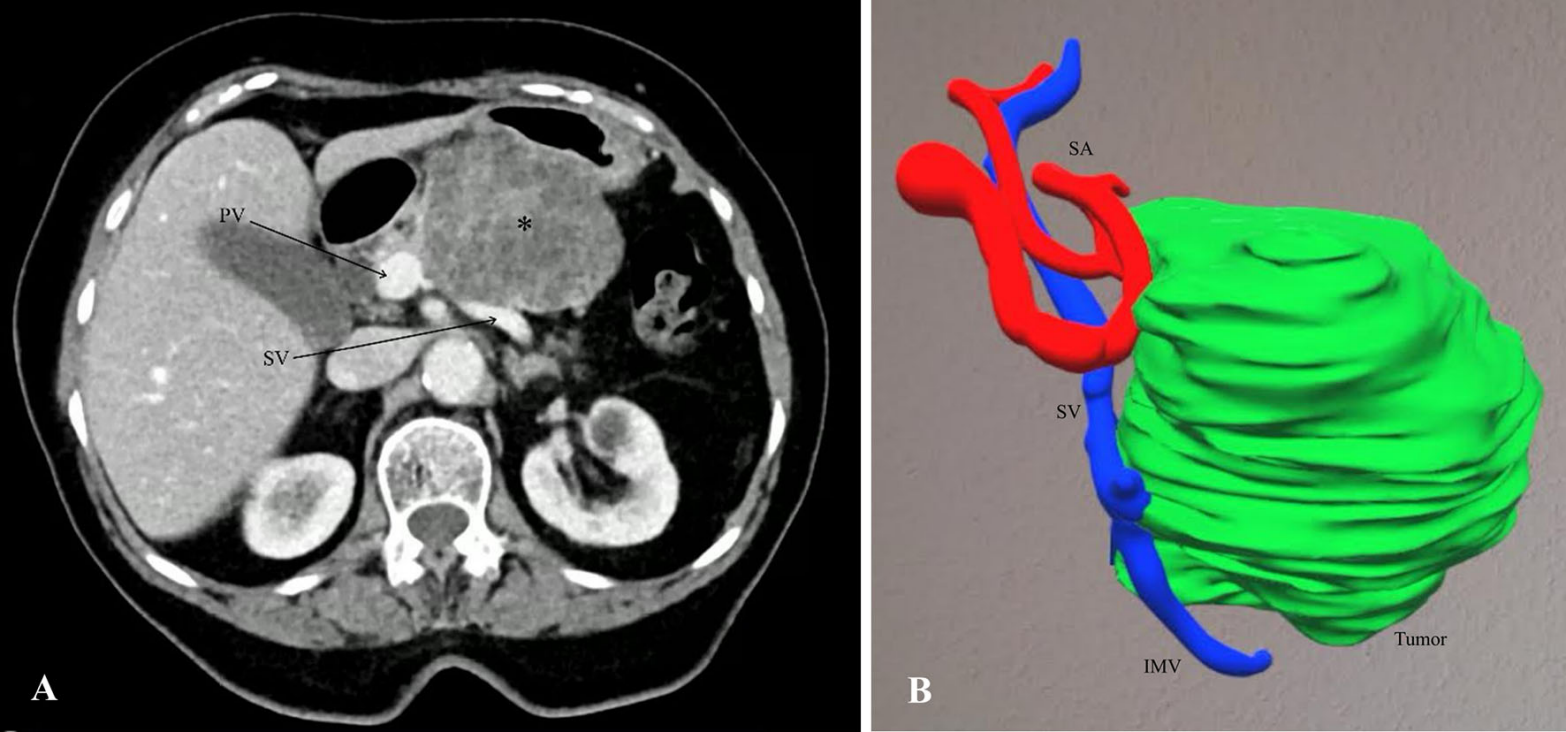
Preoperative planning of the procedure using **a** computed tomography images and **b** 3D model, illustrating the tortuous course of the splenic artery (SA), and proximity of the tumor (*) and splenic vein (SV). Portal vein, PV; inferior mesenteric vein, IMV

82- and 73-year-old female patients were diagnosed with a cystic tumor in the body of the pancreas, demonstrating features of malignancy. Therefore, the surgical resection of the identified lesions was deemed necessary. Preoperative planning was performed using CT images and a reconstructed 3D model of ARAS. Upon reviewing the CT images, spleen-preserving distal pancreatectomy was initially planned for patient 2 (Fig. 5a). However, the reconstructed 3D model revealed a highly tortuous course of the splenic artery along with the proximity of the tumor to the splenic vein. Consequently, ARAS assessment indicated that spleen preservation would be challenging (Fig. 5b). For patient 3, ARAS-guided planning led to the decision to perform extended distal pancreatosplenectomy.

**Fig. 6:**
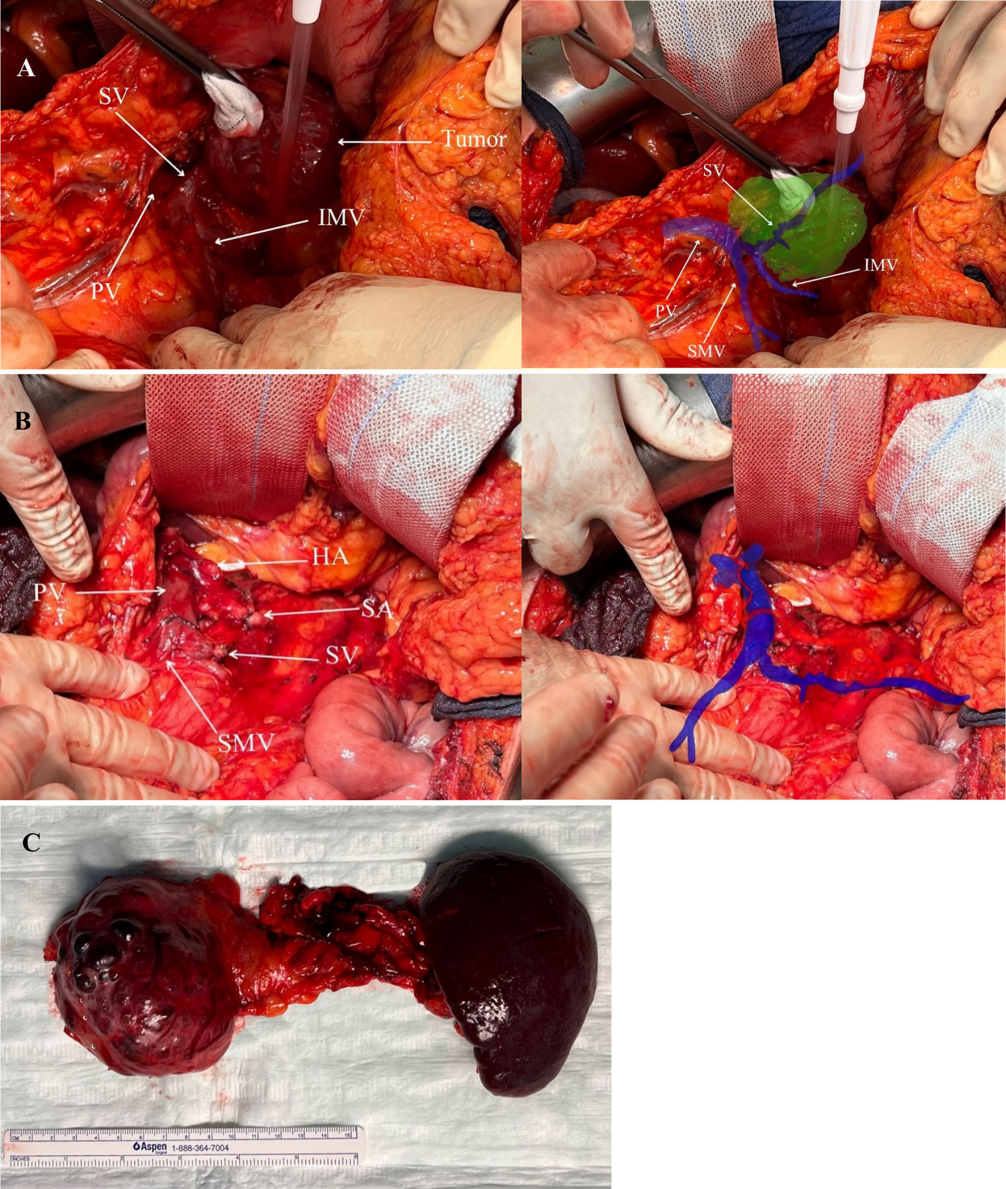
Intraoperative snapshot illustrating **a** the preparation phase using ARAS, **b** identification of vascular system using ARAS after resection phase; **c** resected specimen. Splenic vein, SV; splenic artery, SA; portal vein, PV; hepatic artery, HA; superior mesenteric vein, IMV; inferior mesenteric vein, IMV

No issues were observed intraoperatively during registration of the 3D model. The vascular structures were effectively visualized with the assistance of ARAS (Fig. 6a, Patient 2). In line with the preoperative planning, the operation for patient 2 revealed a notably tortuous course of the splenic artery, and the splenic vein was tightly attached to the tumorous process, necessitating distal pancreatectomy with en bloc splenectomy. Even after the resection phase, all the dissected vessels remained identifiable using ARAS (Fig. 6b, Patient 2). Patient 3 underwent extended distal pancreatosplenectomy as planned, with precise identification of the tumor and vascular systems during the operation.

Both surgeries resulted in minimal intraoperative blood loss (50 mL each) and had durations of 125 and 140 min, respectively. Histopathological examination revealed the presence of serous and mucinous cystic neoplasms with clear resection margins in patients 2 and 3, respectively. Both patients were discharged without complications seven days after the operation.

### Patient 4 and 5 (pancreaticoduodenectomy)

**Fig. 7:**
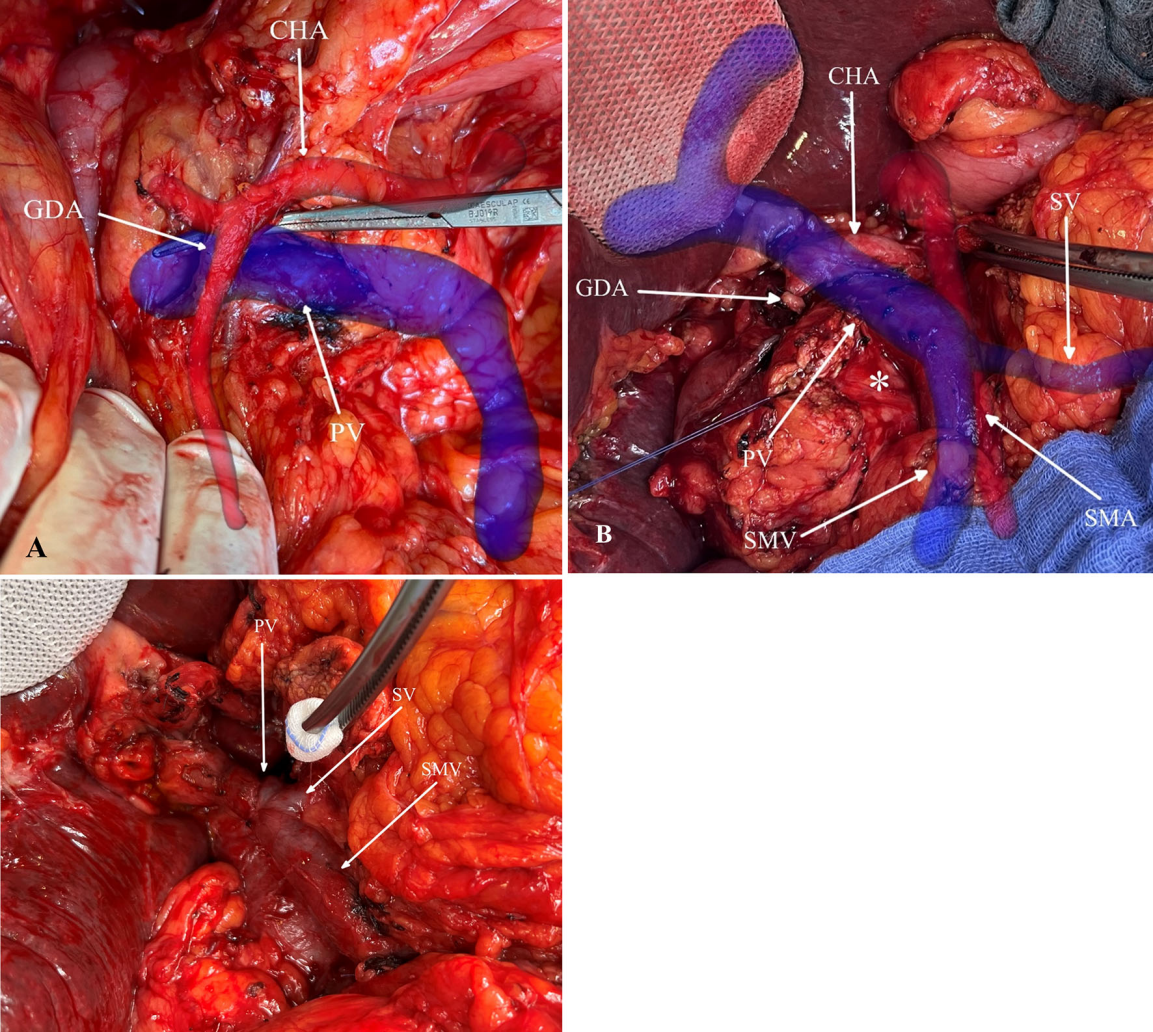
Intraoperative snapshot illustrating **a** the preparation of the gastroduodenal artery (GDA) and portal vein (PV) using ARAS, **b** identification of vascular system and dorsal tumor infiltration into the PV (*) after pancreas transection, and **c** PV reconstruction after resection phase. Common hepatic artery, CHA; splenic vein, SV; superior mesenteric artery, SMA; superior mesenteric vein, SMV

Two patients, a 59-year-old male and a 69-year-old female, diagnosed with uncinate process cancer, underwent preoperative evaluation using ARAS. Subsequently, pylorus-preserving pancreaticoduodenectomy (PPPD) via an uncinate-first approach was planned. The initial registration of the 3D models was successfully completed for both the patients. As shown in Fig. 7a, ARAS played a crucial role in guiding vascular identification and ensuring safe preparation.

However, during the operation in the fifth patient, dorsal tumor infiltration into the portal vein and close proximity of the tumor to the SMA were observed (Fig. 7b). Although ARAS facilitated precise identification and preparation of the SMA, owing to the extent of tumor involvement with the portal vein, PPDD with portal vein resection and subsequent reconstruction were performed (Fig. 7c).

To enhance mobilization and ensure safe preparation of the portal vein and SMA, an uncinate-first approach was employed. However, this approach led to significant deformity and mobilization of the organ. Consequently, the accuracy of tumor registration was compromised during the resection phase.

Intraoperative blood loss was 350 and 400 mL, with operation durations of 310 and 280 min, respectively. Histopathological examination revealed pancreatic adenocarcinoma with R0 resection margins in both the cases. The patients were discharged without any major complications 10 and 12 days after the operation, respectively.

### Assessment of ARAS during pancreatic surgery

In the initial phase of surgery, preceding the preparatory steps, the location and precision of all augmented structures were carefully evaluated through the examination of arterial pulses and/or the utilization of intraoperative Doppler and duplex ultrasonography. Consequently, all the structures were accurately identified without any noteworthy errors, ensuring secure vascular preparation and dissection.

Following the preparation and dissection of adjustment tissues, particularly in PPPD (patients 4 and 5), the identification of the tumor became less reliable owing to the release and shifting of the pancreas. However, the vascular system, notably the GDA, SMA, and portal vein, which are crucial landmarks during pancreatic resection, remained consistently identifiable without significant errors throughout all surgical procedures. Despite the absence of a requirement for ARAS after the resection phase, all dissected vessels remained identifiable through the system.

**Fig. 8:**
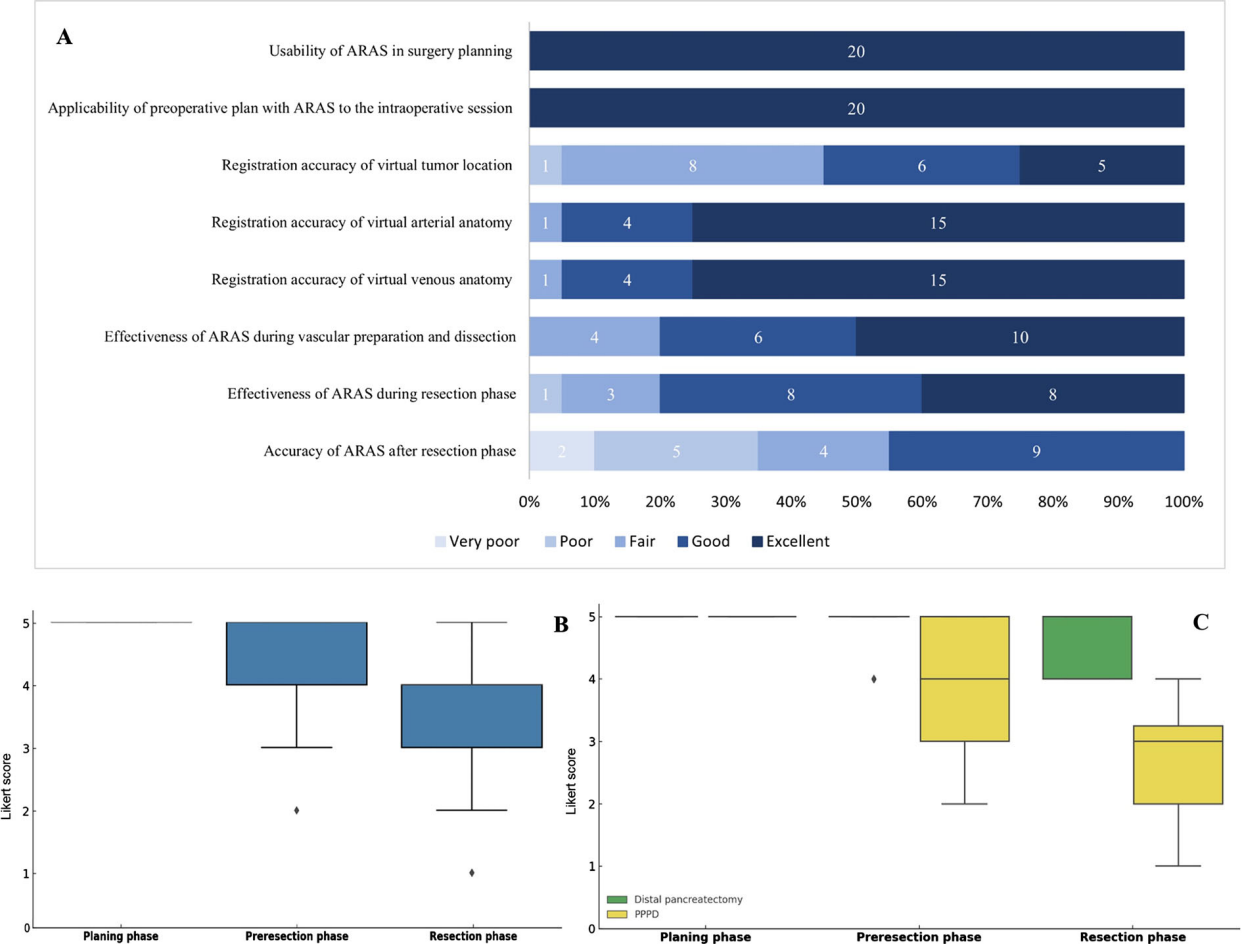
**a** Responses on five-point Likert scale questionnaire (in percent; the figures indicate the frequency per response category; the response categories range from “very poor” (1) to “excellent” (5). **b** The box plot illustrating the assessment of usability, accuracy, and overall effectiveness of ARAS for pre- and intraoperative sessions. **c** Illustration of usability, accuracy, and overall effectiveness of ARAS between two different surgical procedures. Planning phase: Q1–2; preresection phase: Q3–6; resection phase: Q 7–8. Questions 1–8 were also presented in Table 1. Pylorus preserving pancreaticoduodenectomy, PPPD

Furthermore, as illustrated in Fig. 8, surgeons rated the usability of ARAS as excellent (mean score, 5 ± 0) for preoperative planning of the procedure. No deviations from the planned procedures were documented based on preoperative planning facilitated by ARAS. During the pre-resection phase of surgery, ARAS demonstrated precise identification of the vascular system, earning a mean score of 4.4 ± 0.8 as a highly effective guidance system. This accuracy translates into secure preparation and dissection of the vascular system without any recorded complications. However, as anticipated, the reliability of tumor identification decreased after the preparation phase, particularly in patients who underwent PPPD (Fig. 8c; mean score for the resection phase: 4.4 ± 0.2 vs. 2.7 ± 0.8).

## Discussion

The integration of AR technology in the medical field has evolved over the years, spanning from its initial role as an educational and training tool to its current status as a surgical instrument [17, 18]. In particular, its application in orthopedic, plastic, and neurosurgery has demonstrated significant utility as a preoperative and intraoperative aid [6–8, 19]. Studies have highlighted the potential benefits of AR technology, including educational advantages, increased surgical accuracy, reduced radiation exposure, decreased operative time, and improved workload perception by surgeons [6, 8, 19–21]. Despite these advancements, the development of AR-based guidance systems for abdominal surgery faces challenges, primarily due to major organ shifting and deformities [9, 10].

Pancreatic cancer, with its poor prognosis and challenging surgical intricacies, requires innovative approaches to enhance its outcomes. Moreover, the retroperitoneal anatomy of the pancreas suggests minimal intraoperative organ and vascular system displacement and deformation. Accordingly, the present prospective study explored the utilization of wearable AR technology in pancreatic surgery, with the goal of evaluating the usability, accuracy, and effectiveness of the designed ARAS system in enhancing the perioperative outcomes of patients undergoing pancreatic surgery. The present preliminary findings involving five patients underscore the high utility of preoperative surgical planning using ARAS and the consistent and reliable identification of crucial vascular structures during pancreatic surgeries, as confirmed by Doppler and duplex ultrasonography. The remarkable accuracy and effectiveness of this system suggest its substantial contribution to the precision and safety of vascular preparation and dissection.

These findings are consistent with those of the previous studies. Tang et al. [15] developed an AR-based navigation system utilizing a smartphone display during pancreaticoduodenectomy in three patients. Their AR system effectively aided navigation, assisting in the determination of the superior mesenteric vein and tumor invasion area, with an estimated deviation ranging from 2 to 8 mm. Similarly, Marzano [12] and Okamoto et al. [13] employed an AR navigation system in wherein reconstructed 3-D models were superimposed onto real organs using a monitor display. In these investigations, AR facilitated precise and secure identification of the pancreatic organ, tumor location, and critical vascular structures, with a registration error ranging from 5 to 12 mm [15]. Onda et al. [14] conducted an initial comparative study of AR-based and conventional pancreaticoduodenectomy and reported no complications associated with the AR-based guidance system during or after surgery. Although there were no significant differences in operation time and blood loss between the two groups in this study, the AR-based guidance system provided precise anatomical information, enabling the prompt identification and ligation of important vessels.

However, all previous studies employed 2D-display-based modalities with predominantly manual 3D model registration, potentially impeding spatial perception and extending operation durations. In this study, a pioneering approach is presented that introduces a wearable ARAS with a marker-based AR approach along with two distinct tracking methodologies as progressive registration methods specifically designed for pancreatic surgery. The wearable AR approach surpasses display-based AR in surgical assistance systems by reducing the divided attention effect, allowing surgeons to maintain focused attention in the surgical field without the need for a separate display. Furthermore, wearable technologies offer superior depth perception compared to display-based AR in surgical assistance system [22]. By projecting augmented information directly onto the surgeon’s field of view, wearable devices leverage binocular vision and natural eye movement, providing a more accurate and intuitive sense of depth. This enhanced depth perception is crucial in intricate surgical procedures, as it allows surgeons to perceive spatial relationships with greater precision.

The preparation for using ARAS in the operating room for all surgical procedures required only 10 min, involving the immediate registration of 3D models, which could be repeated during the operation as needed. ARAS was used continuously by two surgeons throughout all surgeries without encountering any issues. Intraoperative parameters, including blood loss and operation time, align with our retrospective data and existing literature [23], affirming that the implementation of ARAS did not compromise surgical efficiency.

In this study, the application of ARAS was explored in complex surgical procedures, such as the Appleby procedure and the uncinate process-first approach, which necessitate extensive mobilization of peripancreatic structures. Nonetheless, the decline in the reliability of tumor identification following the preparation phase, especially in patients who underwent PPPD (Fig. 8c), underscores the challenges associated with organ shifting and deformation even during pancreatic surgery. This emphasizes the need for a real-time system that is capable of tracking dynamic anatomical changes during surgical procedures.

Last but not least, further assessment of the questionnaires also revealed that junior surgeons actively involved in preoperative planning and intraoperative use of ARAS reported an improved subjective understanding of the surgical plan, identification of anatomical structures, and potential pitfalls during the resection phase. It can be anticipated that the future holds promise for AR-based assistance systems; it is likely that upcoming fellows will experience a steeper learning curve and reduced stress during surgery through the integration of wearable ARAS.

## Conclusion

The present preliminary outcomes suggest that wearable ARAS hold promise for improving surgical precision during pancreatic procedures. The system’s efficacy in preoperative planning and vascular identification may position it as a valuable tool for pancreatic surgery, as well as an educational resource for inexperienced surgical residents. Further clinical investigations are essential, first, to refine and adapt ARAS, particularly in addressing real-time tracking methods during surgery, and second, to evaluate their impact on perioperative short- and long-term outcomes.

## Data Availability

The anonymized raw data used in this study are available upon request to the Ethics Committee of the Medical Association of Saarland.
